# Cytokine profiles and CD8+ T cells in the occurrence of acute and chronic hepatitis B

**DOI:** 10.3389/fimmu.2022.1036612

**Published:** 2022-10-24

**Authors:** Si Xie, Liu Yang, Xiaoyue Bi, Wen Deng, Tingting Jiang, Yanjie Lin, Shiyu Wang, Lu Zhang, Ruyu Liu, Min Chang, Shuling Wu, Yuanjiao Gao, Hongxiao Hao, Ge Shen, Mengjiao Xu, Xiaoxue Chen, Leiping Hu, Yao Lu, Rui Song, Yao Xie, Minghui Li

**Affiliations:** ^1^ Division of Hepatology, Hepato-Pancreato-Biliary Center, Beijing Tsinghua Changgung Hospital, School of Clinical Medicine, Tsinghua University, Beijing, China; ^2^ Department of Hepatology Division 2, Beijing Ditan Hospital, Capital Medical University, Beijing, China; ^3^ Department of Hepatology Division 2, Peking University Ditan Teaching Hospital, Beijing, China; ^4^ Department of Infectious Disease, Beijing Ditan Hospital, Capital Medical University, Beijing, China

**Keywords:** chronic hepatitis B, acute hepatitis B, cytokines, CD8+ T cells, immune tolerance

## Abstract

**Objective:**

We explore the expression of functional molecules on CD8+ T lymphocytes, cytokines concentration, and their correlation to occurrence of hepatitis B and hepatitis B virus (HBV) desoxyribose nucleic acid (DNA), hepatitis B surface antigen (HBsAg), hepatitis B envelope antigen (HBeAg), and alanine aminotransferase (ALT) in patients infected with HBV.

**Methods:**

This is a single center study. 32 patients with acute hepatitis B (AHB), 30 patients with immune tolerant (IT) phase chronic HBV infected, and 50 patients with chronic hepatitis B (CHB) were enrolled. The activation molecules (CD69) and the apoptosis-inducing molecules (CD178) on surface of CD8+ T lymphocytes were tested by the flow cytometry. Fms-like tyrosine kinase 3 ligand (Flt-3L), interleukin 17A (IL-17A), interferon γ (IFN-γ), and Interferon α2 (IFN-α2) were quantitated by Luminex assay. We use linear regression analysis to analyze their correlations to ALT, HBV DNA, HBsAg, and HBeAg.

**Results:**

The frequency of CD69+CD8+ T lymphocytes in CHB and AHB groups were increased significantly compared with IT group (4.19[3.01, 6.18]% and 4.45[2.93, 6.71]% vs. 3.02[2.17, 3.44]%; *H*=26.207, *P*=0.001; *H*=28.585, *P*=0.002), and the mean fluorescence intensity (MFI) of CD69 in AHB group was significantly higher than IT and CHB groups (27.35[24.88, 32.25] vs. 20.45[19.05, 27.75] and 23.40[16.78, 28.13]; *H*=25.832, *P*=0.005 and *H*=22.056, *P*=0.008). In IT group, HBsAg levels and HBV DNA loads were negatively correlated with CD69MFI (*β*=-0.025, *t*=-2.613, *P*=0.014; *β*=-0.021, *t*=-2.286, *P*=0.030), meanwhile, HBeAg was negatively related to the frequency of CD69+CD8+ T lymphocytes (*β*=-61.306, *t*=-2.116, *P*=0.043). In AHB group, IFN-α2 was positively related to the frequency of CD8+ T lymphocytes (*β*=6.798, *t*=2.629, *P*=0.016); however, in CHB group, IFN-α2 was negatively associated with frequency of CD8+ T lymphocytes (*β*=-14.534, *t*=-2.085, *P*=0.043). In CHB group, HBeAg was positively associated with frequency of CD69+CD8+ T lymphocytes (*β*=43.912, *t*=2.027, *P*=0.048). In AHB group, ALT was positively related to CD69MFI (*β*=35.042, *t*=2.896, *P*=0.007), but HBsAg was negatively related to CD178MFI (*β*=-0.137, *t*=-3.273, *P*=0.003).

**Conclusions:**

The activation of CD8+ T lymphocytes was associated with the occurrence of AHB and CHB. However, due to the insufficient expression of functional molecules of CD8+ T lymphocytes and the depletion of CD8+ T lymphocytes, CHB patients were difficult to recover from HBV infection.

## Introduction

Long-term progression of hepatitis B virus (HBV) infection will lead to liver failure, cirrhosis, and liver cancer, which will bring great threat to patients’ lives and economic situation. The occurrence and development of hepatitis are closely associated with immune environment of hosts and virus after HBV infection (1). HBV infection always persists in majority of chronic hepatitis B (CHB), but can be cleared spontaneously in acute hepatitis B (AHB). We know that there are four phases in the natural outcome of CHB patients depending on different immune states (2, 3). These differences mainly depend on the differential immune responses of patients.

Various immune cells played a role in the immune response of patients with hepatitis B. Innate immunity mediated by natural killer (NK) cells, NKT cells, dendritic cells (DCs), and mononuclear macrophages can inhibit virus spread, participate in antigen presentation and activate specific immunity, which is the first line of antiviral defense. Adaptive immunity, mainly involving B cells, CD8+ T lymphocytes and CD4+ T lymphocytes, is considered to not only inhibit virus replication, but also play an important role in virus clearance. CD8+ T lymphocytes are of crucial importance in HBV clearance. In addition, it is difficult to achieve persistent viral clearance and hepatitis B surface antigen (HBsAg) serological conversion in patients infected with HBV, which may be associated with the deficiency of HBV-specific adaptive immunity (4). This study tried to investigate the function of CD8+ T lymphocytes and related cytokines to illustrate different immune state in HBV infected patients. In HBV infection, CD8+ T lymphocytes can inhibit viral replication by secreting interferon γ (IFN-γ), and induce apoptosis of infected hepatocytes through the secretion of perforin, which causes the rupture of HBV infected hepatocytes and the expression of apoptotic molecules. Thus, CD8+ T lymphocytes are of crucial importance in HBV clearance. The major of adults infected with HBV can recover from infection. The immune response caused by HBV infection leads to severe hepatic inflammation in the liver. However, this immune response in CHB patients is not effective in clearing HBV. Many kinds of immune cells, like NK cells, DCs, mononuclear macrophages, CD4+ T lymphocytes, CD8+ T lymphocytes and their secreted cytokines, are involved in the occurrence of liver inflammation and viral clearance. The outcome of HBV infection depends on the function of HBV-specific cellular immunity. In our research, we investigate the relationship between the immune environment and clinical indicators in patients infected with HBV.

## Materials and methods

### Patients

Eligible patients attending Beijing Ditan Hospital from February 2014 to December 2016 were consecutively enrolled. The enrolled objects in the study were assigned to one of the three subgroups: immune tolerant (IT) phase chronic HBV infection group, hepatitis B envelope antigen (HBeAg)-positive CHB group, and AHB group. IT phase chronic HBV infection was defined as HBsAg positive for > 24 weeks, HBV desoxyribose nucleic acid (DNA) >10^7^IU/ml, HBeAg positive, consistently normal alanine aminotransferase (ALT) (<30 U/L in male, <19 U/L in female), and/or no obvious liver fibrosis and inflammation in histology. CHB was defined as HBsAg positive for > 24 weeks, ALT in abnormal level (>120 U/L) for more than 12 weeks, and/or obvious liver fibrosis and inflammation in histology, with positive HBeAg. AHB was defined as no HBV infection 24 weeks ago, HBsAg positive, abnormal ALT, and anti-HBc-IgM for > 1:1000 or confirmed by histopathological examination (2, 3). Exclusion criteria: co-infected with other hepatitis viruses, co-infected with other viruses, with other liver diseases (such as alcoholic, immune, metabolic), and liver cancer; with cirrhosis or fibrosis diagnosed by transient elastography (5). Patients with autoimmune diseases, and subjects using immunosuppressive or regulatory drugs were also excluded. The liver cancer was excluded by CT examination (Computed Tomography System, 103 LightSpeed VCT, LightSpeed Pro32, Tokyo, Japan) or ultrasound (Acuson Sequoia, Siemens, Erlangen, Germany). The Liver cirrhosis or fibrosis test was taken by FibroScan (FibroScan 502, EchosensTM, Paris, 102 France).

This is a single center study. Our research was approved by Beijing Ditan Hospital Institutional Review Board. All objects have signed informed consent before enrollment.

### Clinical indicators, serology and HBV DNA detection

The biochemical indicators were tested by Hitachi 7600 automatic biochemical analyzer (Hitachi 7600-11; Hitachi, Tokyo, Japan). Virological indicator was measured by Roche Cobas AmpliPrep/Cobas TaqMan 96 automatic real-time fluorescence quantitative polymerase chain reaction detection (PCR) detection reagent (Roche, Pleasanton, CA, USA; with a lower detection limit of 20IU/ml). Serological indexes were quantitated by Abbott Architect i2000 detection reagent (Abbott Diagnostics, USA).

### Functional molecular expression and frequency of CD8+ T lymphocytes

The frequency of CD8+ T lymphocytes of all patients in leukomonocytes, CD69+CD8+ T lymphocytes, CD178+CD8+ T lymphocytes, and the counts of CD69 and CD178 expressed on CD8+ T lymphocytes were detected by flow cytometry (FACS Caliburflow Cytometer, USA). It was performed as follow steps: 1) using monoclonal Antibodies (mAbs) of CD3-peridinin chlorophyll protein (PerCP), CD8-human antigen presenting cells (APC), CD69-phycoerythrin (PE), and CD178-PE (BD Biosciences, Cowley, UK) incubated 100μl of whole peripheral blood samples in the dark for 20 minutes; 2) incubated with 2ml fluorescently activated cell classifier (FACS) lysate for 5 mins. 3) after centrifuged at 300×g 5 minutes and aspirated the supernatant, 2ml phosphate buffered saline (PBS) was added and vortex lightly; 4) after centrifuged at 300×g 5 minutes and aspirated the supernatant, the sample was re-suspended with 200μl of PBS and finally analyzed by FACS flow cytometer. CD8+T lymphocytes were identified as monocytes which were in the subsequent showing CD3+/CD8+. CD3+CD8+CD69+ was considered to be the activated CD8+T lymphocytes, CD3+CD8+CD178+ was considered to be the apoptosis-inducing CD8+T lymphocytes.

### Plasma cytokines quantitation

The serum cytokines were quantitated by Luminex assay widespread using for serum cytokine measure. The plasma was separated from EDTA anticoagulant peripheral venous blood, and at −80°C in refrigerator for the cytokine test. Interferon α2 (IFN-α2), IFN-γ, fms-like tyrosine kinase 3 ligand (Flt-3L), and interleukin 17A (IL-17A) were measured. Each sample was measured duplicate, and the standard cytokine supplied by the manufacturer were run on each plate. The operation process was taken according to manufacturer’s instructions. The cytokine levels were obtained by the data acquired by Luminex assay and analyzed using FlexMap 3D analyzer (Austin, TX, USA).

### Statistical methods

In our study, SPSS 22.0 (SPSS Inc., Chicago, IL, USA) was used for statistical analysis. Categorical variables were expressed as absolute values, and were compared using Fisher’s exact test or Chi-square test between different groups. In this study, we express continuous variables in terms of mean ± standard deviation or median (Q1, Q3). Continuous variables were compared using analysis of variance and independent samples *t*-test or Kruskal-Wallis test between groups, according to whether they are normal distribution. We use linear regression analysis to analyze correlation of CD8+ T lymphocytes with clinical indexes (ALT level, HBsAg level, HBeAg content, and HBV DNA load). It was considered to be statistically significant if *P <*0.05.

## Results

### Clinical features of the patients

112 patients (63 males, 49 females) participated in this research, including 30 patients in IT phase, 32 patients with AHB, and 50 patients with CHB, with median age of 30 (26, 39) years. Significant differences were showed in age (27[25.75, 31.25], 30[27.00, 36.25], 33[27.00, 49.75]y; *H*=7.904, *P*=0.019), ALT levels (29.85[21.83, 39.85], 252.70[129.45, 361.50], 930.10[447.25, 1902.48] U/L; *H*=74.981, *P*=0.000), total bilirubin (TBIL) levels (10.25[7.33, 14.43], 14.10[11.90, 18.15], 52.55[25.48, 111.02] μmol/L; *H*=55.928, *P*=0.000), albumin (ALB) (47.45[45.68, 49.30], 44.90[42.30, 47.00], 42.95[38.05, 44.35] g/L; *H*=28.965, *P*=0.000), HBsAg levels (4.79[4.59, 4.93], 3.89[3.46, 4.19], 2.80[2.18, 3.59] log_10_IU/ml; *H*=60.732, *P*=0.000), HBeAg content (1606.36[1556.53, 1679.44], 933.34[467.38, 1316.67], 5.93[0.92, 59.31] S/CO; *H*=77.956, *P*=0.000), and HBV DNA (8.11[8.11 ± 0.48], 7.05[7.05 ± 1.23], 4.61[4.61 ± 1.40] log_10_IU/ml; *H*=61.988, *P*=0.000) among the three groups (**Table 1**).

**Table 1:** Comparison of clinical characteristics, frequency and functional molecular expression of CD8+ T cells between the three groups.

Values	IT group (n=30)	CHB group (n=50)	AHB group (n=32)	*P*	*P1* (IT vs CHB)	*P2* (IT vs AHB)	*P3* (CHB vs AHB)
Sex (male/female)	14/16	28/22	21/11	*χ^2^=*2.264, *P*=0.322	*χ^2^=*0.655, *P*=0.418	*χ^2^=*2.264, *P*=0.132	*χ^2^=*0.752, *P*=0.386
Age (years) (median [Q1, Q3])	27.00 (25.75, 31.25)	30.00 (27.00, 36.25)	33.00 (27.00, 49.75)	*H*=7.904, *P*=0.019	*H*=13.960, *P*=0.187	*H*=22.988, *P*=0.016	*H*=9.028, *P*=0.656
ALT (U/L) (median [Q1, Q3])	29.85 (21.83, 39.85)	252.70 (129.45, 361.50)	930.10 (447.25, 1902.48)	*H*=74.981, *P*=0.000	*H*=42.435, *P*=0.000	*H*=70.360, *P*=0.000	*H*=27.925, *P*=0.000
TBIL (μmol/L) (median [Q1, Q3])	10.25 (7.33, 14.43)	14.10 (11.90, 18.15)	52.55 (25.48, 111.02)	*H*=55.928, *P*=0.000	*H*=19.529, *P*=0.027	*H*=59.358, *P*=0.000	*H*=39.829, *P*=0.000
ALB (g/L) (median [Q1, Q3])	47.45 (45.68, 49.30)	44.90 (42.30, 47.00)	42.95 (38.05, 44.35)	*H*=28.965, *P*=0.000	*H*=-17.692, *P*=0.047	*H*=-43.774, *P*=0.000	*H*=-26.082, *P*=0.001
HBsAg (log_10_IU/ml) (median [Q1, Q3])	4.79 (4.59, 4.93)	3.89 (3.46, 4.19)	2.80 (2.18, 3.59)	*H*=60.732, *P*=0.000	*H*=-37.003, *P*=0.000	*H*=64.099, *P*=0.000	*H*=-27.096, *P*=0.001
HBeAg (S/CO) (median [Q1, Q3])	1606.36 (1556.53, 1679.44)	933.34 (467.38, 1316.67)	5.93 (0.92, 59.31)	*H*=77.956, *P*=0.000	*H*=-35.893, *P*=0.000	*H*=-72.833, *P*=0.000	*H*=-36.940, *P*=0.000
HBV DNA (log_10_IU/ml) (mean ± SD)	8.11 ± 0.48	7.05 ± 1.23	4.61 ± 1.40	*H*=61.988, *P*=0.000	*H*=-25.993, *P*=0.001	*H*=-63.741, *P*=0.000	*H*=-37.748, *P*=0.000
CD8+/LC (%) (mean ± SD)	25.05 ± 6.31	24.60 ± 7.24	27.11 ± 7.14	*F*=1.333, *P*=0.268	*t*=0.287, *P*=0.775	*t*=-1.201, *P*=0.235	*t*=-1.544, *P*=0.127
CD69+/CD8+ (%) (median [Q1, Q3])	3.02 (2.17, 3.44)	4.19 (3.01, 6.18)	4.45 (2.93, 6.71)	*H*=15.439, *P*=0.000	*H*=26.207, *P*=0.001	*H*=28.585, *P*=0.002	*H*=2.379, *P*=1.000
CD8+CD69MFI (median [Q1, Q3])	20.45 (19.05, 27.75)	23.40 (16.78, 28.13)	27.35 (24.88, 32.25)	*H*=12.195, *P*=0.002	*H*=3.777, *P*=1.000	*H*=25.832, *P*=0.005	*H*=22.056, *P*=0.008
CD178+/CD8+ (%) (median [Q1, Q3])	1.03 (0.83, 1.67)	1.16 (0.85, 1.50)	1.16 (0.90, 1.93)	*H*=1.622, *P*=0.445	*H*=0.714, *P*=0.398	*H*=1.485, *P*=0.223	*H*=0.388, *P*=0.533
CD8+CD178MFI (mean ± SD)	21.00 ± 4.73	22.10 ± 6.05	23.92 ± 5.27	*F*=0.642, *P*=0.528	*t*=-0.846, *P*=0.400	*t*=-2.284, *P*=0.026	*t*=-1.395, *P*=0.167

ALT, Alanine aminotransferase; TBIL, total bilirubin; ALB, albumin; HBsAg, hepatitis B surface antigen; HBeAg, hepatitis B e antigen; HBV DNA, hepatitis B virus desoxyribose nucleic acid; MFI, mean fluorescence intensity; IT, immune tolerance; CHB, chronic hepatitis B; AHB, acute hepatitis B.

### Frequency and functional molecular expression of CD8+ T lymphocytes

**Figure 1:**
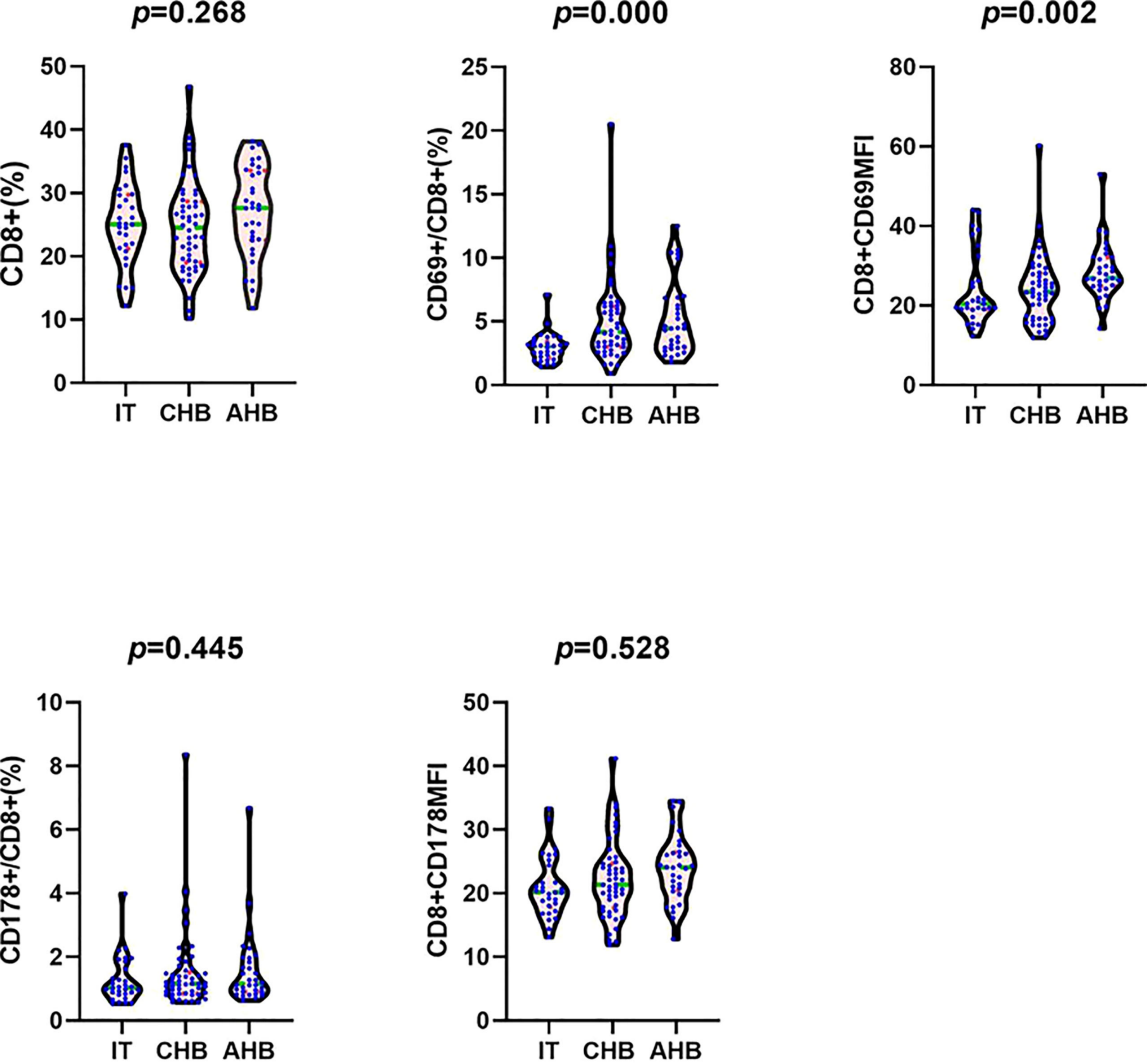
Comparison of the frequency of CD8+ T cells in leukomonocytes, the frequency of CD69+CD8+ T cells in CD8+ T cells, the CD8+CD69MFI, the frequency of CD178+CD8+ T cells in CD8+ T cells, and the CD8+CD178MFI between IT, CHB, and AHB groups. Note: MFI, mean fluorescence intensity; IT, immune tolerance; CHB, chronic hepatitis B; AHB, acute hepatitis B.

Frequency of CD8+ T lymphocytes in leukomonocytes was similar between the three groups. Frequency of CD69+CD8+ T lymphocytes in CHB and AHB groups were increased significantly compared with IT group (4.19[3.01, 6.18] % and 4.45[2.93, 6.71] % vs. 3.02[2.17, 3.44] %; *H*=26.207, *P*=0.001; *H*=28.585, *P*=0.002). The CD69 mean fluorescence intensity (MFI) in AHB group was significantly higher than that in IT and CHB groups (27.35[24.88, 32.25] vs. 20.45[19.05, 27.75] and 23.40[16.78, 28.13]; H=25.832, P=0.005 and H=22.056, P=0.008). Frequency of CD178+CD8+ T lymphocytes and the CD178MFI in CHB and AHB groups were slightly higher than those in IT group (1.16[0.85, 1.50] % and 1.16[0.90, 1.93] % vs. 1.03[0.83, 1.67] %; 22.10 ± 6.05 and 23.9 ± 25.27 vs. 21.00 ± 4.73), but no significant differences (*P*>0.05) were found (**Figure 1** and **Table 1**).

### Correlation of CD8+ T lymphocytes with clinical indexes and cytokines

In IT group, HBsAg and HBV DNA load had negative correlation with CD69MFI (*β*=-0.025, *t*=-2.613, *P*=0.014; *β*=-0.021, *t*=-2.286, *P*=0.030), and HBeAg was negatively correlated with frequency of CD69+CD8+ T lymphocytes (*β*=-61.306, *t*=-2.116, *P*=0.043). In CHB group, HBeAg was positively related to frequency of CD69+CD8+ T lymphocytes (*β*=43.912, *t*=2.027, *P*=0.048), and IFN-α2 was negatively related to frequency of CD8+ T lymphocytes (*β*=-14.534, *t*=-2.085, *P*=0.043). In AHB group, ALT was positively related to CD69MFI (*β*=35.042, *t*=2.896, *P*=0.007), HBsAg was negatively related to CD178MFI (*β*=-0.137, *t*=-3.273, *P*=0.003), and IFN-α2 was positively related to the frequency of CD8+ T lymphocytes (*β*=6.798, *t*=2.629, *P*=0.016) (**Table 2**).

**Table 2:** Correlation of CD8+ T cells with clinical indexes and cytokines.

	IT group					CHB group					AHB group				
	CD8+ (%)	CD69+ (%)	CD69MFI	CD178+ (%)	CD178MFI	CD8+ (%)	CD69+ (%)	CD69MFI	CD178+ (%)	CD178MFI	CD8+ (%)	CD69+ (%)	CD69MFI	CD178+ (%)	CD178MFI
ALT (U/L)	*t*=1.227, *P*=0.230	*t*=-0.777, *P*=0.444	*t*=-0.620, *P*=0.540	*t*=-1.358, *P*=0.185	*t*=1.474, *P*=0.152	*t*=-0.668, *P*=0.508	*t*=-0.638, *P*=0.527	*t*=-0.161, *P*=0.873	*t*=0.094, *P*=0.925	*t*=-0.348, *P*=0.729	*t*=0.752, *P*=0.458	*t*=0.128, *P*=0.899	*t*=2.896, *P*=0.007	*t*=-0.471, *P*=0.641	*t*=1.178, *P*=0.248
HBsAg (log_10_IU/ml)	*t*=0.118, *P*=0.907	*t*=-1.378, *P*=0.179	*t*=-2.613, *P*=0.014	*t*=1.119, *P*=0.273	*t*=0.972, *P*=0.339	*t*=0.713, *P*=0.480	*t*=0.967, *P*=0.338	*t*=-0.345, *P*=0.731	*t*=0.459, *P*=0.648	*t*=-0.969, *P*=0.337	*t*=0.825, *P*=0.416	*t*=1.426, *P*=0.164	*t*=-0.209, *P*=0.836	*t*=-0.233, *P*=0.817	*t*=-3.273, *P*=0.003
HBeAg (S/CO)	*t*=1.659, *P*=0.108	*t*=-2.116, *P*=0.043	*t*=-1.494, *P*=0.146	*t*=-0.112, *P*=0.911	*t*=0.914, *P*=0.369	*t*=1.313, *P*=0.196	*t*=2.027, *P*=0.048	*t*=0.053, *P*=0.958	*t*=-0.021, *P*=0.983	*t*=-0.164, *P*=0.870	*t*=0.672, *P*=0.507	*t*=-0.261, *P*=0.796	*t*=-0.770, *P*=0.447	*t*=0.010, *P*=0.992	*t*=-1.618, *P*=0.116
HBV DNA (log_10_IU/ml)	*t*=-0.712, *P*=0.482	*t*=-0.770, *P*=0.448	*t*=-2.286, *P*=0.030	*t*=1.427, *P*=0.165	*t*=0.567, *P*=0.575	*t*=0.585, *P*=0.561	*t*=1.870, *P*=0.068	*t*=1.210, *P*=0.232	*t*=0.599, *P*=0.552	*t*=-0.561, *P*=0.578	*t*=0.190, *P*=0.851	*t*=-0.191, *P*=0.850	*t*=0.958, *P*=0.346	*t*=-0.519, *P*=0.607	*t*=-0.793, *P*=0.434
Flt-3L (pg/ml)	*t*=-0.202, *P*=0.842	*t*=-0.374, *P*=0.712	*t*=-1.029, *P*=0.315	*t*=1.891, *P*=0.072	*t*=0.050, *P*=0.961	*t*=-1.915, *P*=0.062	*t*=-1.028, *P*=0.310	*t*=-0.409, *P*=0.685	*t*=-1.108, *P*=0.274	*t*=-0.594, *P*=0.556	*t*=0.760, *P*=0.456	*t*=-1.500, *P*=0.149	*t*=-0.473, *P*=0.641	*t*=0.089, *P*=0.930	*t*=-0.960, *P*=0.348
IFN-α2 (pg/ml)	*t*=-0.272, *P*=0.788	*t*=-0.900, *P*=0.378	*t*=-1.080, *P*=0.292	*t*=-0.513, *P*=0.613	*t*=-0.474, *P*=0.640	*t*=-2.085, *P*=0.043	*t*=-0.497, *P*=0.622	*t*=-0.870, *P*=0.389	*t*=-0.776, *P*=0.442	*t*=-0.844, *P*=0.403	*t*=2.629, *P*=0.016	*t*=0.485, *P*=0.633	*t*=1.517, *P*=0.144	*t*=2.025, *P*=0.056	*t*=0.439, *P*=0.665
IFN-γ (pg/ml)	*t*=-0.116, *P*=0.909	*t*=-0.165, *P*=0.870	*t*=-1.471, *P*=0.156	*t*=0.867, *P*=0.395	*t*=-0.994, *P*=0.331	*t*=-1.231, *P*=0.225	*t*=-0.533, *P*=0.597	*t*=-0.511, *P*=0.612	*t*=-0.819, *P*=0.417	*t*=-1.220, *P*=0.229	*t*=0.632, *P*=0.534	*t*=-0.945, *P*=0.355	*t*=-1.004, *P*=0.327	*t*=0.069, *P*=0.946	*t*=-1.574, *P*=0.131
IL-17A (pg/ml)	*t*=0.122, *P*=0.904	*t*=-0.359, *P*=0.723	*t*=-1.424, *P*=0.168	*t*=-0.904, *P*=0.376	*t*=-0.857, *P*=0.400	*t*=-1.230, *P*=0.226	*t*=-0.483, *P*=0.632	*t*=-0.486, *P*=0.630	*t*=-0.699, *P*=0.489	*t*=-1.463, *P*=0.151	*t*=1.440, *P*=0.165	*t*=-0.616, *P*=0.544	*t*=-0.923, *P*=0.367	*t*=-0.032, *P*=0.975	*t*=-1.521, *P*=0.143

ALT, alanine aminotransferase; HBsAg, hepatitis B surface antigen; HBeAg, hepatitis B e antigen; HBV DNA, hepatitis B virus desoxyribose nucleic acid; Flt-3L, fms-like tyrosine kinase 3 ligand; IFN-α2, interferon α2; IFN-γ, interferon γ; IL-17A, interleukin 17A; IT, immune tolerance; CHB, chronic hepatitis B; AHB, acute hepatitis B; MFI, mean fluorescence intensity.

### Correlation of cytokines with clinical parameters

HBV DNA was positively related to Flt-3L, IL-17A and IFN-γ (*β*=0.001, *t*=2.254, *P*=0.034; *β*=0.002, *t*=2.269, *P*=0.033; *β*=0.001, *t*=2.205, *P*=0.038) in IT group. There was no significant correlation between cytokines and HBV DNA, ALT, HBsAg, and HBeAg in CHB patients. In AHB patients, HBeAg was positively related to IL-17A, Flt-3L and IFN-γ (*β*=7.057, *t*=7.763, *P*=0.000; *β*=1.695, *t*=7.123, *P*=0.000; *β*=2.949, *t*=11.940, *P*=0.000), and HBV DNA was also positively related to Flt-3L, IL-17A and IFN-γ (*β*=0.004, *t*=2.343, *P*=0.029; *β*=0.015, *t*=2.320, *P*=0.030; *β*=0.006, *t*=2.671, *P*=0.014) (**Table 3**).

**Table 3:** Correlation of cytokines with clinical indexes.

	IT group				CHB group				AHB group			
	Flt-3L (pg/ml)	IFN-α2 (pg/ml)	IFN-γ (pg/ml)	IL-17A (pg/ml)	Flt-3L (pg/ml)	IFN-α2 (pg/ml)	IFN-γ (pg/ml)	IL-17A (pg/ml)	Flt-3L (pg/ml)	IFN-α2 (pg/ml)	IFN-γ (pg/ml)	IL-17A (pg/ml)
ALT (U/L)	*t*=-0.487, *P*=0.631	*t*=-0.622, *P*=0.540	*t*=0.594, *P*=0.559	*t*=0.347, *P*=0.732	*t*=-0.648, *P*=0.521	*t*=0.255, *P*=0.800	*t*=-0.777, *P*=0.442	*t*=-1.004, *P*=0.321	*t*=0.561, *P*=0.581	*t*=-0.785, *P*=0.441	*t*=-0.010, *P*=0.992	*t*=0.738, *P*=0.469
HBsAg (log_10_IU/ml)	*t*=1.561, *P*=0.133	*t*=0.716, *P*=0.482	*t*=1.088, *P*=0.288	*t*=1.301, *P*=0.207	*t*=-0.347, *P*=0.731	*t*=-1.022, *P*=0.312	*t*=-0.035, *P*=0.972	*t*=0.240, *P*=0.812	*t*=0.942, *P*=0.357	*t*=-0.063, *P*=0.950	*t*=1.855, *P*=0.078	*t*=1.522, *P*=0.143
HBeAg (S/CO)	*t*=0.907, *P*=0.374	*t*=1.200, *P*=0.243	*t*=0.969, *P*=0.343	*t*=1.294, *P*=0.209	*t*=1.328, *P*=0.191	*t*=1.068, *P*=0.292	*t*=1.723, *P*=0.092	*t*=1.726, *P*=0.092	*t*=7.123, *P*=0.000	*t*=0.718, *P*=0.480	*t*=11.940, *P*=0.000	*t*=7.763, *P*=0.000
HBV DNA (log_10_IU/ml)	*t*=2.254, *P*=0.034	*t*=1.403, *P*=0.174	*t*=2.205, *P*=0.038	*t*=2.269, *P*=0.033	*t*=-0.206, *P*=0.838	*t*=-0.783, *P*=0.438	*t*=-0.064, *P*=0.950	*t*=0.109, *P*=0.914	*t*=2.343, *P*=0.029	*t*=-0.188, *P*=0.852	*t*=2.671, *P*=0.014	*t*=2.320, *P*=0.030

ALT, alanine aminotransferase; HBsAg, hepatitis B surface antigen; HBeAg, hepatitis B e antigen; HBV DNA, hepatitis B virus desoxyribose nucleic acid; IT, immune tolerance; CHB, chronic hepatitis B; AHB, acute hepatitis B. Flt-3L, fms-like tyrosine kinase 3 ligand; IFN-α2, interferon α2; IFN-γ, interferon γ; IL-17A, interleukin 17A.

## Discussion

As a non-cytotoxic hepatotropic virus, the clinical manifestation and outcome of HBV infection largely depend on host immunity. In general, a well-developed immune response is likely to induce severe liver injury and viral clearance, so that over 95% patients infecting HBV in adults can recover spontaneously. Conversely, an immature immune response leads to the persistence of HBV, just as the 90% patients infecting HBV in infants become chronic infection (6). HBV-induced immunity is coordinated by innate immunity, specific immunity and cytokines, in which the specific immunity plays an important role in virus clearance (7, 8). Cytotoxic lymphocytes (CTLs) are of crucial importance in HBV-specific immunity. There are rich of HBV-specific CD8+ T lymphocytes in our liver, which can clear HBV by cytosolic or non-cytolytic pathway (9–11). We investigated the function of CD8+ T lymphocytes and related cytokines in HBV infected patients of different states, to explore their role in clinical outcome and to better understand HBV-induced immune pathogenesis.

Specific CD8+ T lymphocytes need to be stimulated by cytokines such as IFN-α to clear HBV. When receiving the stimulation of viral antigen, they secrete cytokines and perforin to inhibit viral replication. And HBV-specific CD8+ T lymphocytes can lead apoptosis of HBV-infected hepatocytes by inducing expression of apoptotic molecules. In our study, we examined major cytokines with virus-clearing effects, expression of CD8+ T lymphocytes activating molecules and apoptosis-inducing molecules in patients with IT, CHB, and AHB patients.

CD69 is the marker of early activated lymphocytes, and it can be up-regulated when lymphocytes are stimulated by antigens. In our study, Patients with AHB and CHB had a significant higher frequency of CD69+CD8+ T lymphocytes compared with IT patients, which may suggest that the occurrence of hepatitis is linked to increased proportion of activated CD8+ T lymphocytes. In HBV infected patients, CD8+ T lymphocytes activated by IFN-α play a role in killing infected hepatocytes (12), and it has been wildly used for immune control and clinical cure of CHB (13, 14). Our research also indicated why interferon therapy is more effective in CHB patients than IT patients, especially in HBV infected patients with severe hepatitis (15, 16). However, although being activated, CD69 molecular expressed on CD8+ T lymphocytes surface just increased slightly in CHB group compared with IT group, but increased significantly in AHB patients compared with IT and CHB patients as shown in our study. This may partly explain why most CHB patients can not eliminate the virus and recover from HBV infection like AHB patients. CD178 (Fas-L) is an apoptosis-inducing molecule on surface of CD8+ T lymphocytes, and can mediate apoptosis of infected liver cells by Fas/Fas-L pathway. In our study, frequency of CD178+CD8+ T lymphocytes and the CD178MFI in AHB and CHB group were slightly higher than those in IT patients. These results indicated that, in addition to mediating the apoptosis of target cells, killing the infected cells directly by secreting cytotoxic molecules like granzyme and perforin, and secreting cytokines involving IFN-γ and TNF-α to inhibit spread and replication of virus are also important ways of CTLs to eliminate HBV (9–11).

It is known that HBV can impair the functions and maturation of DCs (17, 18), and DCs will show tolerance subtypes under long-term stimulation of high levels of HBV and its antigens in CHB (19, 20). In this research, HBV DNA and HBsAg were negatively correlated with CD69MFI, and there was negative correlation between HBeAg level and frequency of CD69+CD8+ T lymphocytes in IT group. These results showed that activation of CD8+ T lymphocytes was also suppressed by long-term high levels of HBV and its antigens. It is consistent with previous researches that the activation of HBV-specific immunity will be affected when innate immune is weakened (21, 22). As mentioned above, IFN-α can stimulate CD8+ T lymphocytes to activate immune response (12). In our study, IFN-α2 was positively related to frequency of CD8+ T lymphocytes in AHB group, which could confirm this statement. Interestingly, we found that IFN-α2 was negatively related to frequency of CD8+ T lymphocytes in CHB group. It may indicate why the HBV-specific CD8+ T lymphocytes exhaust during interferon therapy and lead to a decline in efficacy in CHB group (23, 24).

The outcome of HBV infection is determined by the status of HBV infection and the function of HBV-specific CD8+ T lymphocytes. 95% of adults with acute HBV infection can recover spontaneously, which is correlated with the function of CD8+ T lymphocytes (25). However, it is difficult to recover from chronic HBV infection due to impaired CD8+ T lymphocytes function (26, 27). The response of CD8+ T lymphocytes is also associated with liver inflammation in chronic HBV infection (28). The aim of this study was to investigate the function of CD8+ T lymphocytes in HBV infection, including the expression of activation molecules (CD69) and apoptosis-inducing molecules (CD178) in the pathogenesis of chronic HBV infection (IT and HBeAg-positive CHB), and their role in hepatitis B (CHB and AHB). In our study, patients with CHB had a significant higher frequency of CD69+CD8+ T lymphocytes compared with IT patients, but frequency of CD8+ T lymphocytes, frequency of CD178+CD8+ T lymphocytes and CD178 molecular expressed on CD8+ T lymphocytes surface were all similar between the two groups. Meanwhile, in CHB patients, frequency of CD8+ T lymphocytes, CD69+CD8+ T lymphocytes, CD178+CD8+ T lymphocytes and expression of CD69, CD178 molecules on CD8+ T lymphocytes surface had no significant correlation with HBV DNA load. The results suggested that the activation of CD8+ T lymphocytes might be associated with the onset of hepatitis in chronic HBV infection, but it couldn’t inhibit replication of virus or eliminate infected hepatocytes effectively. This may be related to the presence of high levels of viral antigens in chronic HBV infection, which can suppress the function of HBV-specific CD8+ T lymphocytes (29). And the detection of CD8+ T lymphocytes in peripheral blood may not fully reflect its distribution in liver histology too. We found that frequency of CD8+ T lymphocytes, CD69+CD8+ T lymphocytes and CD178+CD8+ T lymphocytes in AHB patients were also similar to CHB patients. In addition, in AHB patients, frequency of CD8+ T lymphocytes, CD69+CD8+ T lymphocytes, CD178+CD8+ T lymphocytes and expression of CD69, CD178 molecules on CD8+ T lymphocytes surface also had no significant correlation with HBV DNA load. Although the ALT in CHB and AHB groups significantly increased in our study, it might not mainly caused by CD8+ T lymphocytes. Previous studies have found that the hepatitis in chronic HBV infection is associated with the activation and phenotype of NK cells (30). And meta-analysis showed that there was no significant correlation between the function of CD8+ T lymphocytes and ALT level in chronic HBV infection (31). Some studies have found that the inflammatory phase of AHB is also associated with the activation of NK cells (32), and the activation of NK cells may negatively regulate HBV-specific CD8+ T cells (33). Therefore, the occurrence of hepatitis in HBV infection is not only related to the function of CD8+ T lymphocytes, but also related to the function of various immune cells such as NK cells.

Cytokines play a vital role in virus clearance. Cytokines can directly inhibit virus replication, leading necrosis of hepatocytes infected with virus, and stimulate or inhibit other immune cells to conduct immune regulation and form a certain immune environment. We also explored the relativity of cytokines and clinical indexes in this study. Flt-3L also plays an important role as an essential growth factor for NK and DCs (34). IFN-γ is an essential cytokine for CTLs to inhibit HBV replication and eliminate HBV in a non-cytolytic way (35). IL-17A is related to degree of inflammation as a pro-inflammatory cytokine (36). In our study, HBeAg and HBV DNA were positively related to Flt-3L, IL-17A and IFN-γ in AHB patients, which showed the activation of inflammation by HBV. We found that HBV DNA was also positively related to Flt-3L, IL-17A and IFN-γ in IT patients. Although the immune response is deeply downregulated in IT patient, the HBV DNA may still activate mild inflammation.

Our study still had some limitations. First, we only enrolled HBeAg-positive patients in this study for the sake of investigate the correlation of CD8+ T lymphocytes and cytokines with HBV antigens. Second, we didn’t pair baseline parameters among the three groups because it was difficult when patients were consecutively enrolled, which might lead to a bias. Third, the sample was small. Since patients were consecutively enrolled in this study and the sample size was relatively small, it was difficult to handle baseline parameters by propensity score matching. We will conduct large-sample studies with matched baseline parameters in the future to further avoid selection bias. Since occurrence and clearance of hepatitis caused by HBV involve various immune cells and cytokines, the outcome of CHB patients depends on the immune environment. Only some of the cytokines that contribute to CD8+ T lymphocytes function were investigated in this study.

## Conclusion

In conclusion, the activation of CD8+ T lymphocytes was related to occurrence of AHB and CHB. Meanwhile, it is difficult to recover from HBV infection because of its insufficient expression of functional molecules of CD8+ T lymphocytes and depletion of CD8+ T lymphocytes.

## Data availability statement

The raw data supporting the conclusions of this article will be made available by the authors, without undue reservation.

## Ethics statement

The studies involving human participants were reviewed and approved by Institutional Review Board of Beijing Ditan Hospital. The patients/participants provided their written informed consent to participate in this study.

## Author contributions

ML, SX, YaoL, RS, and YX contributed to design and concept. XB, LY, WD, TJ, YanL, SW, LZ, RL, MC, SW, YG, HH, GS, MX, XC, and LH performed the data collection. YX, SX, WD, TJ, YaoL, RS, and ML guided statistical analysis. SX wrote the manuscript. SX checked the revised manuscript. YaoL, RS, YX, and ML modify the version to be submitted. ML submitted the modified version. All authors contributed to the article and approved the submitted version.

## Funding

National Science and Technology Major Project of China(2017ZX10201201-001-006, 2018ZX10715-005-003-005,2017ZX10201201-002-006); The capital health research and development of special(2022-1-2172); Beijing Hospitals Authority Clinical medicine Development of special funding support(XMLX 202127); The Digestive Medical Coordinated Development Center of Beijing Hospitals Authority (XXZ0302,XXT28); Project supported by Beijing science and technology commission(Z211100002921059).

## Conflict of interest

The authors declare that the research was conducted in the absence of any commercial or financial relationships that could be construed as a potential conflict of interest.

The reviewer LJ declared a shared parent affiliation with the authors to the handling editor at the time of review.

## Publisher’s note

All claims expressed in this article are solely those of the authors and do not necessarily represent those of their affiliated organizations, or those of the publisher, the editors and the reviewers. Any product that may be evaluated in this article, or claim that may be made by its manufacturer, is not guaranteed or endorsed by the publisher.

## References

[1] Li MH Lu Y Zhang L Wang XY Ran CP Hao HX. Association of cytokines with alanine aminotransferase, hepatitis b virus surface antigen and hepatitis b envelope antigen levels in chronic hepatitis b. Chin Med J (2018) 131(15):1813–8. doi: 10.4103/0366-6999.237394 PMC6071474 30058578

[2] Terrault NA Lok A McMahon BJ Chang KM Hwang JP Jonas MM. Update on prevention, diagnosis, and treatment of chronic hepatitis b: AASLD 2018 hepatitis b guidance. Clin liver Dis (2018) 12(1):33–4. doi: 10.1002/cld.728 PMC6385899 30988907

[3] Vittal A Ghany MG. WHO guidelines for prevention, care and treatment of individuals infected with HBV: A US perspective. Clinics liver Dis (2019) 23(3):417–32. doi: 10.1016/j.cld.2019.04.008 PMC9616205 31266617

[4] Bertoletti A Tan AT Gehring AJ. HBV-specific adaptive immunity. Viruses (2009) 1(2):91–103. doi: 10.3390/v1020091 21994540 PMC3185487

[5] European Association for Study of Liver Asociacion Latinoamericana para el Estudio del Higado. EASL-ALEH clinical practice guidelines: Non-invasive tests for evaluation of liver disease severity and prognosis. J Hepatol (2015) 63(1):237–64. doi: 10.1016/j.jhep.2015.04.006 25911335

[6] Prendergast AJ Klenerman P Goulder PJ. The impact of differential antiviral immunity in children and adults. Nat Rev Immunol (2012) 12(9):636–48. doi: 10.1038/nri3277 22918466

[7] Ferrari C. HBV and the immune response. Liver international: Off J Int Assoc Study Liver (2015) 35 Suppl 1:121–8. doi: 10.1111/liv.12749 25529097

[8] Fisicaro P Valdatta C Boni C Massari M Mori C Zerbini A. Early kinetics of innate and adaptive immune responses during hepatitis b virus infection. Gut (2009) 58(7):974–82. doi: 10.1136/gut.2008.163600 19201769

[9] Guidotti LG. The role of cytotoxic T cells and cytokines in the control of hepatitis b virus infection. Vaccine (2002) 20 Suppl 4:A80–2. doi: 10.1016/s0264-410x(02)00392-4 12477433

[10] He JS Gong DE Ostergaard HL. Stored fas ligand, a mediator of rapid CTL-mediated killing, has a lower threshold for response than degranulation or newly synthesized fas ligand. J Immunol (Baltimore Md. (2010) 1950) 184(2):555–63. doi: 10.4049/jimmunol.0902465 19949069

[11] Shivakumar P Mourya R Bezerra JA. Perforin and granzymes work in synergy to mediate cholangiocyte injury in experimental biliary atresia. J Hepatol (2014) 60(2):370–6. doi: 10.1016/j.jhep.2013.09.021 PMC3946990 24096050

[12] Lambotin M Raghuraman S Stoll-Keller F Baumert TF Barth H. A look behind closed doors: interaction of persistent viruses with dendritic cells. Nat Rev Microbiol (2010) 8(5):350–60. doi: 10.1038/nrmicro2332 PMC2988821 20372157

[13] Piratvisuth T Marcellin P Popescu M Kapprell HP Rothe V Lu ZM. Hepatitis b surface antigen: association with sustained response to peginterferon alfa-2a in hepatitis b e antigen-positive patients. Hepatol Int (2013) 7(2):429–36. doi: 10.1007/s12072-011-9280-0 21701902

[14] Li MH Zhang L Qu XJ Lu Y Shen G Li ZZ. The predictive value of baseline HBsAg level and early response for HBsAg loss in patients with HBeAg-positive chronic hepatitis b during pegylated interferon alpha-2a treatment. Biomed Environ Sci BES (2017) 30(3):177–84. doi: 10.3967/bes2017.025 28427487

[15] Sonneveld MJ Hansen BE Piratvisuth T Jia JD Zeuzem S Gane E. Response-guided peginterferon therapy in hepatitis b e antigen-positive chronic hepatitis b using serum hepatitis b surface antigen levels. Hepatol (Baltimore Md.) (2013) 58(3):872–80. doi: 10.1002/hep.26436 23553752

[16] Li MH Zhang L Qu XJ Lu Y Shen G Wu SL. Kinetics of hepatitis b surface antigen level in chronic hepatitis b patients who achieved hepatitis b surface antigen loss during pegylated interferon alpha-2a treatment. Chin Med J (2017) 130(5):559–65. doi: 10.4103/0366-6999.200554 PMC5339929 28229987

[17] Lan S Wu L Wang X Wu J Lin X Wu W. Impact of HBeAg on the maturation and function of dendritic cells. Int J Infect Dis IJID Off Publ Int Soc Infect Dis (2016) 46:42–8. doi: 10.1016/j.ijid.2016.03.024 27044523

[18] Hatipoglu I Ercan D Acilan C Basalp A Durali D Baykal AT. Hepatitis b virus e antigen (HBeAg) may have a negative effect on dendritic cell generation. Immunobiology (2014) 219(12):944–9. doi: 10.1016/j.imbio.2014.07.020 25150150

[19] Bertoletti A Ferrari C. Innate and adaptive immune responses in chronic hepatitis b virus infections: towards restoration of immune control of viral infection. Gut (2012) 61(12):1754–64. doi: 10.1136/gutjnl-2011-301073 22157327

[20] Li X Wang Y Chen Y. Cellular immune response in patients with chronic hepatitis b virus infection. Microbial pathogenesis (2014) 74:59–62. doi: 10.1016/j.micpath.2014.07.010 25128091

[21] Li J Han Y Jin K Wan Y Wang S Liu B. Dynamic changes of cytotoxic T lymphocytes (CTLs), natural killer (NK) cells, and natural killer T (NKT) cells in patients with acute hepatitis b infection. Virol J (2011) 8:199. doi: 10.1186/1743-422X-8-199 21535873 PMC3096949

[22] Busca A Kumar A. Innate immune responses in hepatitis b virus (HBV) infection. Virol J (2014) 11:22. doi: 10.1186/1743-422X-11-22 24507433 PMC3922976

[23] Micco L Peppa D Loggi E Schurich A Jefferson L Cursaro C. Differential boosting of innate and adaptive antiviral responses during pegylated-interferon-alpha therapy of chronic hepatitis b. J Hepatol (2013) 58(2):225–33. doi: 10.1016/j.jhep.2012.09.029 23046671

[24] Li MH Xie S Bi XY Sun FF Zeng Z Deng W. An optimized mode of interferon intermittent therapy help improve HBsAg disappearance in chronic hepatitis b patients. Front Microbiol (2022) 13:960589. doi: 10.3389/fmicb.2022.960589 36110295 PMC9468551

[25] Maini MK Boni C Ogg GS King AS Reignat S Lee CK. Direct ex vivo analysis of hepatitis b virus-specific CD8(+) T cells associated with the control of infection. Gastroenterology (1999) 117(6):1386–96. doi: 10.1016/s0016-5085(99)70289-1 10579980

[26] Baudi I Kawashima K Isogawa M. HBV-specific CD8+ T-cell tolerance in the liver. Front Immunol (2021) 12:721975. doi: 10.3389/fimmu.2021.721975 34421926 PMC8378532

[27] Boni C Fisicaro P Valdatta C Amadei B Di Vincenzo P Giuberti T. Characterization of hepatitis b virus (HBV)-specific T-cell dysfunction in chronic HBV infection. J Virol (2007) 81(8):4215–25. doi: 10.1128/JVI.02844-06 PMC1866111 17287266

[28] Maini MK Boni C Lee CK Larrubia JR Reignat S Ogg GS. The role of virus-specific CD8(+) cells in liver damage and viral control during persistent hepatitis b virus infection. J Exp Med (2000) 191(8):1269–80. doi: 10.1084/jem.191.8.1269 PMC2193131 10770795

[29] Peng G Luo B Li J Zhao D Wu W Chen F. Hepatitis b e-antigen persistency is associated with the properties of HBV-specific CD8 T cells in CHB patients. J Clin Immunol (2011) 31(2):195–204. doi: 10.1007/s10875-010-9483-5 21120686

[30] Dunn C Brunetto M Reynolds G Christophides T Kennedy PT Lampertico P. Cytokines induced during chronic hepatitis b virus infection promote a pathway for NK cell-mediated liver damage. J Exp Med (2007) 204(3):667–80. doi: 10.1084/jem.20061287 PMC2137916 17353365

[31] Zheng J Ou Z Xu Y Xia Z Lin X Jin S. Hepatitis b virus-specific effector CD8+ T cells are an important determinant of disease prognosis: A meta-analysis. Vaccine (2019) 37(18):2439–46. doi: 10.1016/j.vaccine.2019.03.058 30935741

[32] Webster GJ Reignat S Maini MK Whalley SA Ogg GS King A. Incubation phase of acute hepatitis b in man: dynamic of cellular immune mechanisms. Hepatol (Baltimore Md.) (2000) 32(5):1117–24. doi: 10.1053/jhep.2000.19324 11050064

[33] Peppa D Gill US Reynolds G Easom NJ Pallett LJ Schurich A. Up-regulation of a death receptor renders antiviral T cells susceptible to NK cell-mediated deletion. J Exp Med (2013) 210(1):99–114. doi: 10.1084/jem.20121172 23254287 PMC3549717

[34] Guimond M Freud AG Mao HC Yu J Blaser BW Leong JW. *In vivo* role of Flt3 ligand and dendritic cells in NK cell homeostasis. J Immunol (Baltimore Md. 1950) (2010) 184(6):2769–75. doi: 10.4049/jimmunol.0900685 PMC2924750 20142363

[35] Guidotti LG Ishikawa T Hobbs MV Matzke B Schreiber R Chisari FV. Intracellular inactivation of the hepatitis b virus by cytotoxic T lymphocytes. Immunity (1996) 4(1):25–36. doi: 10.1016/s1074-7613(00)80295-2 8574849

[36] Montes M Zhang X Berthelot L Laplaud DA Brouard S Jin J. Oligoclonal myelin-reactive T-cell infiltrates derived from multiple sclerosis lesions are enriched in Th17 cells. Clin Immunol (Orlando Fla.) (2009) 130(2):133–44. doi: 10.1016/j.clim.2008.08.030 PMC6961709 18977698

